# Canonical Hamiltonian ensemble representation of dephasing dynamics and the impact of thermal fluctuations on quantum-to-classical transition

**DOI:** 10.1038/s41598-021-89400-3

**Published:** 2021-05-11

**Authors:** Hong-Bin Chen, Yueh-Nan Chen

**Affiliations:** 1 Department of Engineering Science, National Cheng Kung University, Tainan, 70101 Taiwan; 2 Center for Quantum Frontiers of Research and Technology, NCKU, Tainan, 70101 Taiwan; 3 Department of Physics, National Cheng Kung University, Tainan, 70101 Taiwan

**Keywords:** Quantum information, Quantum mechanics, Theoretical physics, Quantum physics, Qubits

## Abstract

An important mathematical tool for studying open quantum system theory, which studies the dynamics of a reduced system, is the completely positive and trace-preserving dynamical linear map parameterized by a special parameter-time. Counter-intuitively, akin to the Fourier transform of a signal in time-sequence to its frequency distribution, the time evolution of a reduced system can also be studied in the frequency domain. A recent proposed idea which studies the representation of dynamical processes in the frequency domain, referred to as canonical Hamiltonian ensemble representation (CHER), proved its capability of characterizing the noncalssical traits of the dynamics. Here we elaborate in detail the theoretical foundation within a unified framework and demonstrate several examples for further studies of its properties. In particular, we find that the thermal fluctuations are clearly manifested in the manner of broadening CHER, and consequently rendering the CHER less nonclassical. We also point out the discrepancy between the notions of nonclassicality and non-Markovianity, show multiple CHERs beyond pure dephasing, and, finally, to support the practical viability, propose an experimental realization based upon the free induction decay measurement of nitrogen-vacancy center in diamond.

## Introduction

The ubiquity of the open quantum theory has attracted many attentions among the quantum physics community, with applications ranging from physics, chemistry, to biology^1–9^, note to mention its fundamental importance in the development of frontier technologies^10–12^. Irrespective of the unitary time evolution of the total system–environment arrangement, the reduced system dynamics typically exhibits an incoherent behaviour after dropping the inaccessible ambient environment. One of the main causes of such incoherent behaviour stems from the loss of information on the system–environment correlations, which comes from the interactions between the system and its ambient environment, and significantly modulate the properties of the reduced system dynamics, e.g., from Markovianity to non-Markovianity^13–18^.

Generically, such incoherent dynamics are described in terms of a family of *time*-parametrized completely positive and trace-preserving (CPTP) maps acting on the system density matrices^19–22^. However, since the set of CPTP maps does not form a well-characterized algebraic structure, its mathematical characterization is nontrivial; consequently, it stimulates the development of several different, but intimately related, techniques for characterizing CPTP maps, including operator-sum representation, Kraus operators^23^, process matrices^24^, and Choi–Jamiołkowski isomorphism^25,26^.

In addition, to derive appropriate equations of motion governing the open system dynamics, one typically adopts an assumption of Born–Markov approximation, which leads to the master equation in the standard Lindblad form^27,28^. However, practical problems, e.g., with strong system–environment interactions and/or when long-lived environmental correlations play significant roles, usually do not meet this assumption. To go beyond the Born–Markov regime while taking the memory effects into account, many efforts have been devoted to the construction of improved techniques, such as path-integral formalisms^29–31^, hierarchy equations of motion (HEOM)^32–34^, the reaction-coordinate method^35,36^, and non-Markovian quantum master equations^37–39^.

In the aforementioned approaches, the dynamics of a reduced system has been studied along the time-axis; nevertheless, conventional time evolutions can be studied from a brand new viewpoint. Let us first recall that Fourier transform, which transforms signals in time domain to a frequency domain, enables one to study and manipulate the characters of the signal in the frequency domain. Then, a similar picture emerges where a bosonic field can be described with the Wigner function^40^ or the Glauber–Sudarshan *P* representations^41,42^. These are (quasi-)distribution functions over certain corresponding phase spaces, rather than real spaces, and are capable of characterizing the nonclassical features of the boson field. On the other hand, even a century after the birth of quantum theory, the essential quantum nature and the question of distinguishing the genuine quantum traits from classical counterparts remain contentious and intriguing due to the fundamental importance. In particular, the boundary between quantum and classical realm and how classical characters emerge from quantum essence have given rise to vast debates^43–46^.

Inspired by the above insights, we have proposed to describe a unital dynamics with a (quasi-)distribution function over frequency domain, referred to as the canonical Hamiltonian ensemble representation (CHER)^47,48^. In order to acquire additional properties of the CHER, exploring its versatility in characterizing the nonclassicality of unital dynamics, and the impact of thermal fluctuations, here we first elaborate in detail the theoretical foundation within a unified framework and recast it into Fourier transform formalism. We then demonstrate several examples of qubit pure dephasing dynamics with or without revealing nonclassical traits. We conclude that, generically, increasing the environmental temperature broadens the CHERs and leads to shallower negative wings, i.e., diminishment of nonclassical traits. This agrees with the usual intuition that thermal fluctuations are detrimental to the quantum nature and constitute one of the primary origins of quantum-to-classical transitions. Furthermore, by varying the Ohmicity of the spectral density and the environmental temperature, we can observe a transition between Markovianity and non-Markovianity as well as competition between non-Markovian memory effect and Markovian thermal fluctuations. These phenomena can be easily understood from the deformation of the CHERs. Our results suggest that the notion of nonclassicality is different from the non-Markovianity. Further studies on distinguishing the two notions are highly desired.

We have also discussed the uniqueness of CHER for pure dephasing dynamics, which is underpinned by the abelian algebraic structure of the Hamiltonian ensemble (HE). Therefore, one can expect the breakdown of uniqueness when going beyond pure dephasing. To explicitly show this breakdown of uniqueness, we consider an example of general qubit unital dynamics. Based on the approach we have established, one has redundant freedom in constructing the CHER for unital dynamics. Upon arriving at a CHER for a unital dynamics, one can arbitrarily generate more CHERs by adding high order spherical harmonics with $l\ge 3$ to the known one. Notably, these CHERs lead to the same qubit unital dynamics. We therefore draw the conclusion that there are multiple representations for unital dynamics.

Finally, we have elucidated a promising experimental proposal based on the free induction decay measurement of the electron spin associated with a diamond defect. Due to the three-order difference between spin qubit relaxation time $T_1$ and dephasing time $T_2^*$, the qubit dynamics can be well approximated by pure dephasing. Therefore, the dynamical behaviour and the corresponding CHER can be determined experimentally by using a variant of Ramsey pulse sequence. This circumvents the burden of performing the standard quantum process tomography experiment, meanwhile underpinning the practical viability and the compatibility of CHER theory with present-day techniques.

## Results

### Averaged dynamics under HE

**Figure 1:**
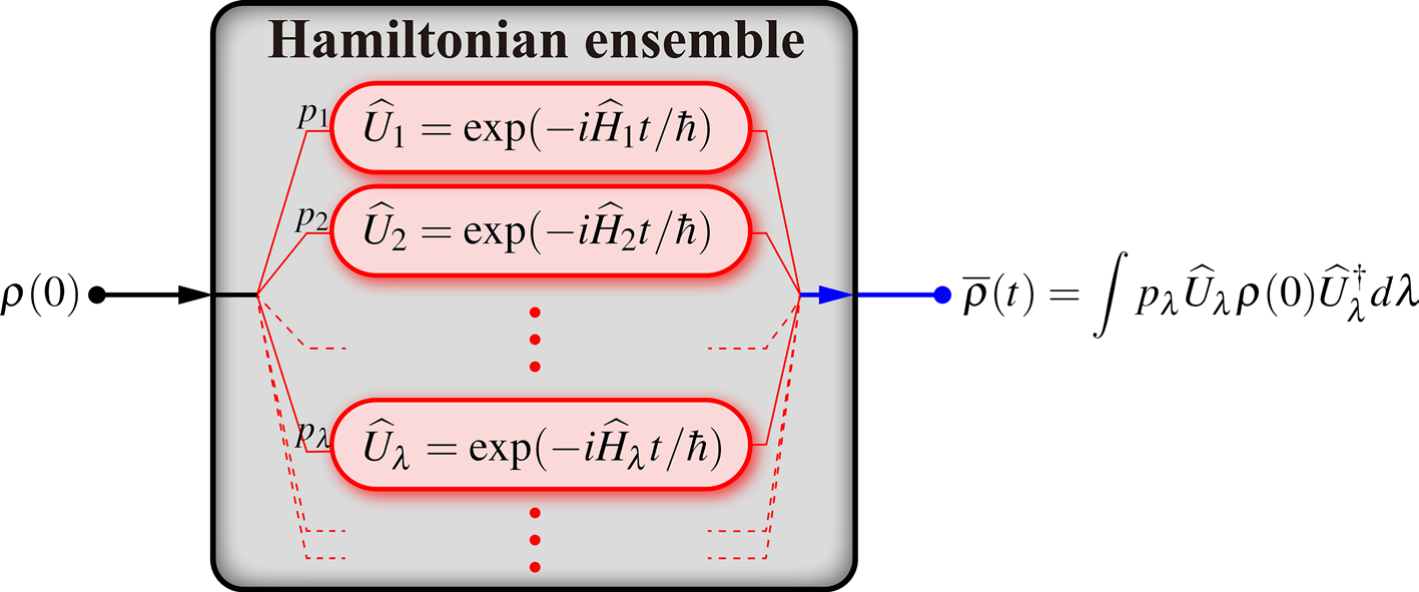
Schematic illustration of a HE. A HE $\{(p_\lambda ,\widehat{H}_\lambda )\}_\lambda$ consists of a collection of Hermitian operators $\widehat{H}_\lambda$ and a probability distribution $p_\lambda$ of occurrence. In each single run of an experiment, the initial state $\rho (0)$ is randomly assigned to a unitary channel $\widehat{U}_\lambda =\exp (-i\widehat{H}_\lambda t/\hbar )$, denoted by red boxes, according to $p_\lambda$. Finally, all the output states are mixed together, leading to an ensemble-averaged dynamics given by Eq. (1).

The mathematical tool of fundamental importance in this work is the Hamiltonian ensemble (HE). A HE $\{(p_\lambda ,\widehat{H}_\lambda )\}_\lambda$ consists of a collection of (time-independent) Hermitian operators $\widehat{H}_\lambda$ of the same dimension, and a probability distribution $p_\lambda$ of occurrence. The index $\lambda$ is generic and may be continuous and/or a multi-index. Each member Hamiltonian $\widehat{H}_\lambda$ generates a unitary time-evolution operator $\widehat{U}_\lambda =\exp (-i\widehat{H}_\lambda t/\hbar )$. For each single run of an experiment, we input an initial state $\rho (0)$ into the HE, which randomly assigns $\rho (0)$ to a unitary channel $\widehat{U}_\lambda$ according to the probability distribution $p_\lambda$ (Fig. 1). The ensemble-averaged dynamics after many runs is given by the unital map

1 $$\begin{aligned} \overline{\rho }(t)=\mathcal {E}_t\{\rho (0)\}=\int p_\lambda \widehat{U}_\lambda \rho (0)\widehat{U}_\lambda ^\dagger d\lambda . \end{aligned}$$

Due to the averaging procedure over all unitary realizations, the time-evolved state $\overline{\rho }(t)$ undergoes a dephasing dynamics and behaves incoherently^49–52^.

An instructive example^50^ is on a single qubit subject to spectral disorder with HE given by $\{(p(\omega ),\hbar \omega {\hat{\sigma }}_z/2)\}_\omega$; i.e., all the member Hamiltonian operators are proportional to ${\hat{\sigma }}_z$ with fluctuating energy level spacing $\hbar \omega$ and $p(\omega )$ can be any probability distribution function. The resulting dynamics is pure dephasing

2 $$\begin{aligned} \overline{\rho }(t)=\int _{-\infty }^\infty p(\omega )e^{-i\omega {\hat{\sigma }}_zt/2}\rho _0e^{i\omega {\hat{\sigma }}_zt/2}d\omega =\left[ \begin{array}{cc} \rho _{\uparrow \uparrow } &{} \rho _{\uparrow \downarrow }\phi (t) \\ \rho _{\downarrow \uparrow }\phi ^*(t) &{} \rho _{\downarrow \downarrow } \end{array} \right] , \end{aligned}$$

with the dephasing factor $\phi (t)=\int p(\omega )e^{-i\omega t}d\omega$ being the Fourier transform of $p(\omega )$.

**Figure 2:**
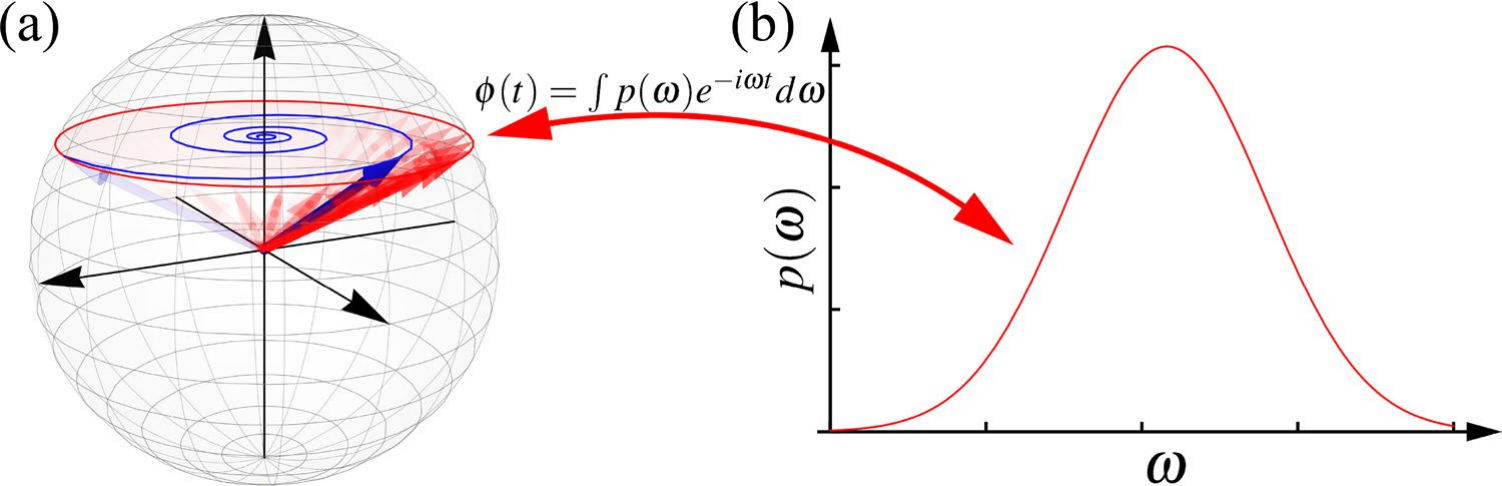
(**a**) Random phase model of qubit pure dephasing. The ensemble of red arrows denotes the random unitary rotations generated by $\hbar \omega {\hat{\sigma }}_z/2$. The opacity reflects that each of the unitary operators $\widehat{U}_\omega$ is weighted by the probability distribution $p(\omega )$. The ensemble-averaged state, denoted by the blue arrow, then undergoes a pure dephasing dynamics. (**b**) The probability distribution function $p(\omega )$ fully determines the pure dephasing dynamics via the Fourier transform; consequently, it is promoted to a representation of qubit pure dephasing in frequency domain.

Since each time-evolution operator $\widehat{U}_\omega =\exp (-i\omega {\hat{\sigma }}_zt/2)$ has a geometric interpretation, namely, an unitary rotation about the *z*-axis of the Bloch sphere at an angular velocity $\omega$, the above HE, as well as the resulting pure dephasing dynamics (2), can be schematically illustrated in terms of random phase (Fig. 2). The ensemble of red arrows in Fig. 2a denotes the random unitary rotations generated by the member Hamiltonian operators $\hbar \omega {\hat{\sigma }}_z/2$ weighted by the probability distribution $p(\omega )$. The ensemble average results in the blue arrow, whose dynamical behaviour is pure dephasing due to the random phase of the ensemble of red arrows. Consequently, such HE-description of pure dephasing agrees with the conventional interpretation of random phase and capable of providing further insights into the algebraic structure behind the dephasing dynamics^48^.

Additionally, whenever the probability distribution function $p(\omega )$ is specified, the pure dephasing dynamics (2) is fully determined. This implies that, as illustrated in Fig. 2b, one can use $p(\omega )$ to characterize the qubit pure dephasing dynamics, and can be recovered by the Fourier transform in Eq. (2). Particularly, as $p(\omega )$ is distributee over the angular velocity $\omega$ and is related to the pure dephasing via the Fourier transform, it becomes a representation of qubit pure dephasing in the frequency domain. It is worth noting that the concept of HE is originally proposed to describe the influence of a disordered environment^49,50^; whereas the experimental realizations of the controlled dephasing are achieved in a similar spirit of statistical mixture^53–55^, and the revival of quantum correlations in the absence of back-action has also been studied in terms of similar ensemble description^56,57^.

### Canonical Hamiltonian-ensemble representation

Exploration of the full power of the (quasi-)probability representation in the frequency domain, referred to as CHER, and its ability to characterize the nonclassical nature of dephasing dynamics is undertaken here.

In the case of *n*-dimension, both member Hamiltonian operators $\widehat{H}_\lambda$ and density matrices $\rho$ are Hermitian and belong to the Lie algebra $\mathfrak {u}(n)=\mathfrak {u}(1)\oplus \mathfrak {su}(n)$. Every $\widehat{H}_\lambda \in \mathfrak {u}(n)$ is a linear combination

3 $$\begin{aligned} \widehat{H}_\lambda =\lambda _0\widehat{I}+\sum _{m=1}^{n^2-1} \lambda _m\widehat{L}_m=\lambda _0\widehat{I}+\vec {\lambda }\cdot \widehat{\varvec{L}} \end{aligned}$$

of identity operator $\widehat{I}$ and $n^2-1$ traceless Hermitian generators $\widehat{L}_m$ of $\mathfrak {su}(n)$; and so does $\rho$. Therefore, the index $\lambda =\{\lambda _0,\vec {\lambda }\}$ parameterizing the HE consists of two components, $\lambda _0\in \mathbb {R}$ and $\vec {\lambda }=\{\lambda _m\}_m\in \mathbb {R}^{n^2-1}$. In order to highlight the role of the representation of a time evolution in the frequency domain played by $p_\lambda$ encapsulated within the HE, we will recast Eq. (1) into a similar form to Fourier transform. This is achieved with the help of several Lie algebra techniques.

To investigate further, we begin with

4 $$\begin{aligned} \exp \left( -i\widehat{H}_\lambda t\right) \rho \exp \left( i\widehat{H}_\lambda t\right) =\sum _{\mu =0}^\infty \frac{(-it)^\mu }{\mu !}\left[ \widehat{H}_\lambda ,\rho \right] _{(\mu )}, \end{aligned}$$

where we have set $\hbar =1$ so that $\lambda$ is in unit of angular frequency, and the multiple commutators are defined as

5 $$\begin{aligned}&\left[ \widehat{H}_\lambda ,\rho \right] _{(0)}=\rho , \end{aligned}$$

6 $$\begin{aligned}&\left[ \widehat{H}_\lambda ,\rho \right] _{(1)}=\left[ \widehat{H}_\lambda ,\rho \right] , \end{aligned}$$

and

7 $$\begin{aligned} \left[ \widehat{H}_\lambda ,\rho \right] _{(\mu )}=\left[ \widehat{H}_\lambda ,\left[ \widehat{H}_\lambda ,\rho \right] _{(\mu -1)}\right] . \end{aligned}$$

Since every density matrix $\rho =n^{-1}\widehat{I}+\vec {\rho }\cdot \widehat{\varvec{L}}$ enjoys the same linear combination as Eq. (3), the commutator in Eq. (6) is determined by $n^2-1$ components $[\widehat{H}_\lambda ,\widehat{L}_m]=\widehat{M}_{\lambda ,m}\in \mathfrak {su}(n)$ as

8 $$\begin{aligned} \left[ \widehat{H}_\lambda ,\rho \right] =\sum _{m=1}^{n^2-1}\rho _m\widehat{M}_{\lambda ,m} =\vec {\rho }\cdot \widehat{\varvec{M}}_\lambda . \end{aligned}$$

Note that $[\widehat{H}_\lambda ,\widehat{I}]=0$. Furthermore, as each $\widehat{M}_{\lambda ,m}$ is also an element in $\mathfrak {su}(n)$, it can be expanded as a linear combination as well:

9 $$\begin{aligned} \widehat{M}_{\lambda ,m}=\vec {h}_{\lambda ,m}\cdot \widehat{\varvec{L}}, \end{aligned}$$

where each $\vec {h}_{\lambda ,m}$ is an $(n^2-1)$-dimensional real vector for $m=1,\ldots ,n^2-1$.

Accordingly, the action of the commutator $[\widehat{H}_\lambda ,\quad ]$ can be conceived as an endomorphism in the sense that it maps a generator $\widehat{L}_m\in \mathfrak {su}(n)$ to the other element $\widehat{M}_{\lambda ,m}\in \mathfrak {su}(n)$ and $\widehat{I}$ to 0; consequently, we can uniquely associate each member Hamiltonian $\widehat{H}_\lambda$ with a linear map $\widetilde{H}_\lambda :\mathfrak {u}(n)\rightarrow \mathfrak {u}(n)$, referred to as the adjoint representation of $\widehat{H}_\lambda$, whose action can be expressed in terms of usual matrix multiplication:

10 $$\begin{aligned} \left[ \widehat{H}_\lambda ,\rho \right] \Rightarrow \widetilde{H}_\lambda \{\rho \}= \left[ \begin{array}{c|ccc} 0 &{} 0 &{} \cdots &{} 0 \\ \hline 0 &{} &{} &{} \\ \vdots &{} \vec {h}_{\lambda ,1} &{} \cdots &{} \vec {h}_{\lambda ,n^2-1} \\ 0 &{} &{} &{} \end{array} \right] \cdot \left[ \begin{array}{c} n^{-1} \\ \hline \\ \vec {\rho } \\ \\ \end{array} \right] . \end{aligned}$$

It is straightforward to see that the multiple commutator in Eq. (7) can be expressed in terms of the adjoint representation $[\widehat{H}_\lambda ,\rho ]_{(\mu )}\Rightarrow \widetilde{H}_\lambda ^\mu \{\rho \}$, as well as Eq. (4) in the adjoint representation

11 $$\begin{aligned} \exp \left( -i\widehat{H}_\lambda t\right) \rho \exp \left( i\widehat{H}_\lambda t\right) \Rightarrow \sum _{\mu =0}^\infty \frac{(-it)^\mu }{\mu !}\widetilde{H}_\lambda ^\mu \{\rho \}=\exp (-i\widetilde{H}_\lambda t)\{\rho \}. \end{aligned}$$

Along with above equations, for a given time-independent HE $\{(\wp _\lambda ,\widehat{H}_\lambda )\}_\lambda$, we can recast the right hand side of Eq. (1) into a Fourier transform expression from a (quasi-)distribution $\wp _\lambda$, on a locally compact group $\mathcal {G}$ parameterized by $\lambda =\{\lambda _0,\vec {\lambda }\}$, to the dynamical linear map $\mathcal {E}_t^{(\widehat{L})}$^48^:

12 $$\begin{aligned} \mathcal {E}_t^{(\widetilde{L})}=\int _\mathcal {G}\wp _\lambda e^{-i\lambda \widetilde{L}t}d\lambda . \end{aligned}$$

Then the action of $\mathcal {E}_t$ on a density matrix $\rho$ can be expressed in terms of usual matrix multiplication

13 $$\begin{aligned} \mathcal {E}_t\{\rho \}\Rightarrow \mathcal {E}_t^{(\widehat{L})}\cdot \rho . \end{aligned}$$

Note that the $\rho$ on the left hand side of Eq. (13) is an $n\times n$ density matrix, while the one on the right hand side is an $n^2$-dimensional real vector $\rho =\{n^{-1},\vec {\rho }\}$. The equation is valid in the sense of the linear combination $\rho =n^{-1}\widehat{I}+\vec {\rho }\cdot \widehat{\varvec{L}}$.

Equation (12) associates a (quasi-)distribution $\wp _\lambda$ with the dynamical process $\mathcal {E}_t$,

14 $$\begin{aligned} \wp _\lambda \mapsto \mathcal {E}_t^{(\widetilde{L})}, \end{aligned}$$

via the Fourier transform on group formalism. This manifests that the role of $\wp _\lambda$ as a CHER for $\mathcal {E}_t$. Moreover, in the above formalism, we have replaced $p_\lambda$ with $\wp _\lambda$ to incorporate the possibility that $\wp _\lambda$ may contain negative values. The underlying physical meaning is an indicator of the nonclassical nature in the dynamical process $\mathcal {E}_t$. This will be clarified in the following discussion.

### Virtual classical environment

Suppose that a system $\rho$ undergoes an averaged dynamics governed by a HE $\{(p_\lambda ,\widehat{H}_\lambda )\}_\lambda$ with $p_\lambda$ being a legitimate probability distribution. Equation (1) has implicitly assumed that the system is isolated in the sense that, beside the uncertainty described by $p_\lambda$, there is no other redundant environmental degree of freedom. However, Eq. (1) results in an incoherent dynamical behaviour, which arises as a consequence of the ensemble average. This is reminiscent of open quantum systems^50^, where the incoherent properties are caused by the interaction to the environment.

Now we can further strengthen this connection between a HE and an open system dynamics by fabricating a virtual environment for a HE encapsulating a legitimate probability distribution $p_\lambda$. Let $\{|\lambda \rangle \}_\lambda$ be an orthonormal basis for the environmental Hilbert space and $\rho _{\rm E}=\int p_\lambda |\lambda \rangle \langle \lambda |d\lambda$ be the virtual environment, then the averaged dynamics in Eq. (1) coincides with the open system dynamics, $\mathcal {E}_t\{\rho (0)\}={\rm Tr}_{\rm E}\rho _{\rm T}(t)$, reduced from the total system

15 $$\begin{aligned} \rho _{\rm T}(t)=\widehat{U}_{\rm T} \left[ \rho (0)\otimes \rho _{\rm E}\right] \widehat{U}^\dagger _{\rm T} \end{aligned}$$

by tracing over the environmental Hilbert space. The total unitary operator $\widehat{U}_{\rm T}=\int \exp (-i\widehat{H}_\lambda t)\otimes |\lambda \rangle \langle \lambda |d\lambda$ is generated by the total Hamiltonian $\widehat{H}_{\rm T}=\int \widehat{H}_\lambda \otimes |\lambda \rangle \langle \lambda |d\lambda$ acting both on the system and the environmental Hilbert spaces.

Additionally, it is noteworthily that the system and the virtual environment in $\rho _{\rm T}(t)$ are at most classically correlated during the evolution, without establishing quantum discord^58,59^. Consequently, the effect of the uncertainty $p_\lambda$ in the HE can be resembled by fabricating a virtual environment, which is classically correlated to the system during the evolution.

### Process nonclassicality

On the other hand, for a genuine quantum system, the inevitable interaction with its environment and the resulting bipartite correlations established during their evolution constitute the primary cause of the incoherent behaviour. As a result, one naive way to characterize the incoherent dynamical processes is based on the properties of the system–environment correlations. Nevertheless, as the environment typically consists of a hug degrees of freedom and cannot be fully accessed in most practical situations, this prevents the viability of this naive approach.

Inspired by the analogy between HE and a classical environment and to circumvent the practical difficulties, we propose an alternative definition of process (non)classicality according to the (im)possibility to simulate the open system with a legitimate HE^47^. The underlying idea of our definition is to deliberately ignore the inaccessible actual environment and try to interpret its effects classically in terms of HE-simulation; meanwhile, the definition relies only on the properties of the system properties, irrespective of the inaccessible environment.

For a given dynamical process $\mathcal {E}_t$, whenever one is able to simulate it with a legitimate HE, i.e., its CHER in Eq. (12) is a legitimate probability distribution, $\mathcal {E}_t$ admits a classical interpretation of mixture of random rotations, and we call such $\mathcal {E}_t$ classical-like, irrespective of actual system–environment correlations. On the other hand, if its CHER necessarily contains negative values, this witnesses the establishment of quantum correlations during the evolution, and the dynamics is nonclassical. Further rigorous proof is shown in Ref. ^47^, relying on the technique of group theory.

### CHER of qubit pure dephasing reduced from spin-boson model

To explicitly exemplify the concept of CHER and the impact of thermal fluctuations, we consider the spin-boson model with total Hamiltonian

16 $$\begin{aligned} \widehat{H}_{\rm T}=\widehat{H}_{\rm S}+\widehat{H}_{\rm E}+\widehat{H}_{\rm I}, \end{aligned}$$

where the system Hamiltonain $\widehat{H}_{\rm S}=\hbar \omega _0{\hat{\sigma }}_z/2$, the environment Hamiltonian $\widehat{H}_{\rm E}=\sum _{\vec {k}}\hbar \omega _{\vec {k}}\hat{b}_{\vec {k}}^\dagger \hat{b}_{\vec {k}}$, and the interaction Hamiltonian $\widehat{H}_{\rm I}={\hat{\sigma }}_z\otimes \sum _{\vec {k}}\hbar (g_{\vec {k}}\hat{b}_{\vec {k}}^\dagger +g_{\vec {k}}^*\hat{b}_{\vec {k}})$. Assuming that the environment is in a thermal equilibrium state at temperature *T*, then this model can be analytically solved^1^ and the qubit dynamics exhibits pure dephasing with the dephasing factor

17 $$\begin{aligned} \phi (t)=\exp [-i\omega _0t-\Phi (t)], \end{aligned}$$

where $\Phi (t)=4\int _0^\infty [\mathcal {J}(\omega )/\omega ^2]\coth (\hbar \omega /2k_{\rm B}T)(1-\cos \omega t)d\omega$ incorporates the information for the interaction and the environmental density of states in terms of the spectral density $\mathcal {J}(\omega )=\sum _{\vec {k}}|g_{\vec {k}}|^2\delta (\omega -\omega _{\vec {k}})$.

In view of Eq. (2), the CHER of the qubit pure dephasing can be obtained from the inverse Fourier transform

18 $$\begin{aligned} \wp (\omega )=\frac{1}{2\pi }\int _{-\infty }^\infty \phi (t)e^{i\omega t}dt, \end{aligned}$$

with respect to the diagonal member Hamiltonian operators $\hbar \omega {\hat{\sigma }}_z/2$ in the simulating HE $\{(\wp (\omega ),\hbar \omega {\hat{\sigma }}_z/2)\}_\omega$. Note that the effect of $\omega _0$ is merely to shift $\wp (\omega )$. Therefore, we can, without loss of generality, assume that $\omega _0=0$ from this position on.

To be a legitimate probability distribution, the resulting CHER $\wp (\omega )$ (18) is expected to satisfy the following conditions:

1. Normalization to unity: $\int _{-\infty }^\infty \wp (\omega )d\omega =1$.
2. Real function: $$\wp (\omega )\in \mathbb {R}, \forall \omega \in \mathbb {R}$$.
3. Positivity: $$\wp (\omega )\ge 0, \forall \omega \in \mathbb {R}$$.

Normalization C1 follows straightforwardly from the fact that $\phi (0)=1$; i.e., $\int _{-\infty }^\infty \wp (\omega )d\omega =(2\pi )^{-1}\int _{-\infty }^\infty \phi (t)2\pi \delta (t-0)dt=1$. Since one typically considers the dynamical behaviour of $\phi (t)$ only for $t\ge 0$, we can freely extend the time domain to all $t\in \mathbb {R}$ such that $\Phi (t)$ is even and $\phi (-t)=\phi (t)^*$. Then $\wp (\omega )=\pi ^{-1}\int _0^\infty \exp [-\Phi (t)]\cos \omega tdt$ is guaranteed to be real, fulfilling condition C2.

While conditions C1 and C2 are straightforward consequences, it cannot be seen immediately whether the condition C3 is fulfilled by $\wp (\omega )$ due to the sinusoidally oscillating behaviour of the integrand in Eq. (18). To prove the positivity of $\wp (\omega )$ for the qubit pure dephasing reduced from the spin-boson mdoel (16), we turn to Bochner’s theory^60^ for help. Further details are given in "Methods". One of our crucial conclusions, the validity of $\wp (\omega )$, is described as follows:

#### Proposition 1

*Suppose that the qubit pure dephasing dynamics characterized by* $\phi (t)=\exp \left[ -i\omega _0 t-\Phi (t)\right]$ *is CP. There exists a unique Hamiltonian ensemble of diagonal member Hamiltonian operators* $\{(\wp (\omega ),\hbar \omega {\hat{\sigma }}_z/2)\}_\omega$ *which can simulate the system dynamics. Additionally, the CHER* $\wp (\omega )=(2\pi )^{-1}\int _{-\infty }^\infty \phi (t)\exp (i\omega t)dt$ *obtained by the inverse Fourier transform of* $\phi (t)$ *is a legitimate probability distribution, satisfying the three conditions for a probability distribution function that are listed above.*

**Figure 3:**
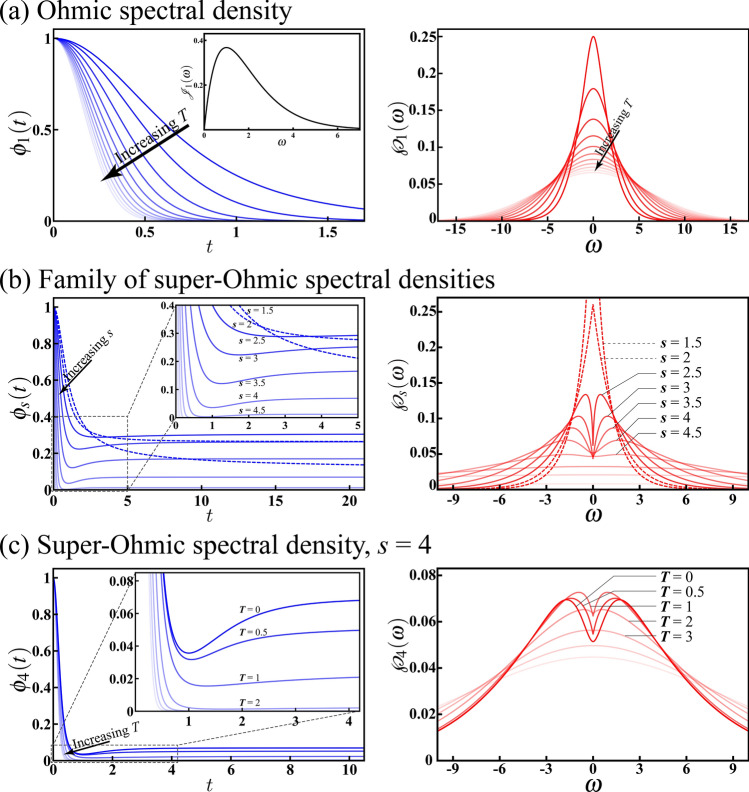
Dynamical behaviour (left panels) and CHER (right panels) of the spin-boson model with various spectral densities. (**a**) The Ohmic spectral density $\mathcal {J}_1(\omega )$ (inset) results in a Markovian pure dephasing as $\phi _1(t)$ decreases monotonically. The increasing *T* reduces the coherence time, while the corresponding $\wp _1(\omega )$ broadens with increasing *T*. In these plots, we have assumed $\omega _{\rm{c}}=1$, $k_{\rm {B}}/\hbar =1$, and increasing *T* from 0 to 5 (decreasing opacity). (**b**) The family of super-Ohmic spectral densities $\mathcal {J}_s(\omega )$ may result in either Markovian (dashed curves) or non-Markovian (solid curves) pure dephasing depending on the Ohmicity *s*, as indicated in the inset. Interestingly, this dynamical transition between Markovian and non-Markovian pure dephasing can also be understood in terms of the deformation of $\wp _s(\omega )$. In these plots, we have assumed $\eta =1/3$, $\omega _{\rm {c}}=1$, and *s* ranging from 1.5 to 6.5 (decreasing opacity). (**c**) Ohmicity $s=4$ is considered and a dynamical transition of $\phi _4(t)$ with increasing *T* from 0 to 5 (decreasing opacity) can be observed, reflecting the fact that the thermal fluctuations will wash out the non-Markovian memory effect. Meanwhile, the competition between the non-Markovian memory effect and the Markovian thermal fluctuations can be observed in terms of the uneven deformation of $\wp _4(\omega )$ with increasing *T*.

In the following, we consider several heuristic examples of different spectral densities. We have also assumed a degenerate qubit such that $\omega _0=0$ in these examples. The resulting $\wp (\omega )$’s are therefore centered at $\omega =0$. Earlier study^1^ shows that the dephasing factor can be split into the vacuum and thermal contributions as $\phi (t)=\exp \left[ -\Phi ^{({\rm vac})}(t)-\Phi ^{({\rm th})}(t)\right]$, where The impact of thermal fluctuations is taken into account by $\Phi ^{({\rm th})}(t)$. Further detailed calculations are given in "Methods".

We first consider the Ohmic spectral density $\mathcal {J}_1(\omega )=\omega \exp (-\omega /\omega _{\rm c})$, as shown in the inset of Fig. 3a, where $\omega _{\rm c}$ is the cut-off frequency. The dephasing factor is given by

19 $$\begin{aligned} \phi _1(t)=\frac{1}{\left( 1+\omega _{\rm c}^2t^2\right) ^2}\prod _{n=1}^\infty \left[ 1 +\left( \frac{\omega _{\rm c}k_{\rm B}T}{k_{\rm B}T+n\hbar \omega _{\rm c}}\right) ^2t^2\right] ^{-4}, \end{aligned}$$

with $k_{\rm B}$ being the Boltzmann constant. It can be seen that Eq. (19) reproduces the case of zero-temperature limit $\phi _1(t)=\left( 1+\omega _{\rm c}^2t^2\right) ^{-2}$ as $T\rightarrow 0$^47^. In the case of finite temperature *T*, the CHER $\wp _1(\omega )=(2\pi )^{-1}\int _{-\infty }^\infty \phi _1(t)\exp (i\omega t)dt$ can only be calculated numerically. The numerical results are shown in Fig. 3a with $\omega _{\rm c}=1$, $k_{\rm B}/\hbar =1$, and increasing *T* from 0 to 5 (decreasing opacity). The left panel of Fig. 3a shows the dynamical behaviour of $\phi _1(t)$. It exhibits a Markovian pure dephasing as $\phi _1(t)$ decreases monotonically; meanwhile, as expected, the coherence time decreases with increasing *T*. The right panel of Fig. 3a shows the corresponding $\wp _1(\omega )$, which broadens when increasing *T*, reflecting stronger thermal fluctuations.

We next consider the family of super-Ohmic spectral densities $\mathcal {J}_s(\omega )=\eta \omega ^s\omega ^{1-s}_{\rm c}\exp (-\omega /\omega _{\rm c})$ parameterized by the coupling strength $\eta$ and the Ohmicity $s>1$. In the zero-temperature limit, the dephasing factor is exclusively given by the vacuum contribution $\phi _s(t)=\exp \left[ -\Phi ^{({\rm vac})}_s(t)\right]$ with

20 $$\begin{aligned} \Phi ^{({\rm vac})}_s(t)=2\eta \Gamma (s-1)\left[ 2-\frac{\left( 1+i\omega _{\rm c}t\right) ^{s-1} +\left( 1-i\omega _{\rm c}t\right) ^{s-1}}{\left( 1+\omega _{\rm c}^2t^2\right) ^{s-1}}\right] , \end{aligned}$$

where $\Gamma (z)$ is the gamma function. The dynamical behaviours of $\phi _s(t)$’s are shown in the left panel of Fig. 3b with $\eta =1/3$, $\omega _{\rm c}=1$, and *s* ranging from 1.5 to 6.5 (decreasing opacity). A transition from Markovianity to non-Markovainity can be seen with increasing *s*. As clearly indicated in the inset, for $1<s\le 2$ (dashed curves), the qubit exhibits a Markovian trait as $\phi _s(t)$’s decrease monotonically. On the other hand, for $s>2$ (solid curves), the qubit pure dephasing is definitely non-Markovian due to the revivals following the initial rapid descents. We stress that the dynamics is always non-Markovian for $s>2$; however the revivals are negligibly small for $s>4.5$. These results are compatible with a previous study ^61^. The numerical results of $\wp _s(\omega )$’s are shown in the right panel of Fig. 3b with increasing Ohmicity *s* from 1.5 to 6.5 (decreasing opacity). The aforementioned transition in the dynamical behaviour manifests itself dramatically in terms of the shape of $\wp _s(\omega )$. Particularly, when *s* is large, $\phi _s(t)$ drops off sharply, therefore the corresponding $\wp _s(\omega )$ gradually flattens. The underlying reason lies in the fact that the varying of the curvature of $\phi _s(t)$ will significantly modulate the shape of its Fourier transform $\wp _s(\omega )$.

In the case of finite temperature *T*, the expression of the dephasing factor $\phi _s(t)=\exp \left[ -\Phi _s(t)\right]$ is complicated due to the presence of thermal fluctuations:

21 $$\begin{aligned} \Phi _s(t)= & {} -2\eta \Gamma (s-1)\left[ 2-\frac{\left( 1+i\omega _{\rm c}t\right) ^{s-1} +\left( 1-i\omega _{\rm c}t\right) ^{s-1}}{\left( 1+\omega _{\rm c}^2t^2\right) ^{s-1}}\right] \nonumber \\&+4\eta \Gamma (s-1)\left( \frac{k_{\rm B}T}{\hbar \omega _{\rm c}}\right) ^{s-1} \left[ 2\zeta \left( s-1,\frac{k_{\rm B}T}{\hbar \omega _{\rm c}}\right) -\zeta \left( s-1,\frac{k_{\rm B}T}{\hbar \omega _{\rm c}}(1+i\omega _{\rm c}t)\right) -\zeta \left( s-1,\frac{k_{\rm B}T}{\hbar \omega _{\rm c}}(1-i\omega _{\rm c}t)\right) \right] , \end{aligned}$$

where $\zeta (s,q)=\sum _{n=0}^\infty (q+n)^{-s}$ is the Hurwitz zeta function. The impact of thermal fluctuations is shown in Fig. 3c with $\eta =1/3$, $\omega _{\rm c}=1$, Ohmicity $s=4$, and increasing *T* from 0 to 5 (decreasing opacity). In the left panel, the dynamical behaviour of $\phi _4(t)$ exhibits a transition from non-Markovianity to Markovianity with increasing *T*, reflecting the fact that the thermal fluctuations will wash out the memory effect and lead to a Markovian dynamics. The right panel shows the corresponding $\wp _4(\omega )$, which generically tends to broaden with increasing *T*. However, it is interesting to note that the aforementioned dynamical transition causes an uneven deformation of $\wp _4(\omega )$, revealing a competition between non-Markovian memory effect and Markovian thermal fluctuations. Additionally, by further comparing the three right panels of Fig. 3, we can qualitatively understand that the effects of thermal fluctuations and increasing *s* are different, even though they both broaden the CHERs.

We have seen several examples of qubit pure dephasing dynamics admitting HE-simulation, i.e., positive CHER. This does not imply the absence of quantum correlations during the evolution. We stress that the qubit does establish entanglement with its environment^62–65^; however, it is not easy to witness its emergence if the total system evolves autonomously without further manipulation. Consequently, its effects are considered classically in terms of HE-simulation and resulting positive CHER. On the other hand, given the above examples, one may question the nonexistence of HE-simulation for other types of qubit pure dephasing dynamics. However, to prove the failure of HE-simulation is, in general, a nontrivial task. In the following, we will study a counterexample of biased spin-boson model leading to CHER showing negative values.

### Nonclassicality of biased spin-boson model

The most general form of interaction Hamiltonian leading to qubit pure dephasing is given by

22 $$\begin{aligned} \widehat{H}_{\rm I}=\sum _{j=\uparrow ,\downarrow }|j\rangle \langle j|\otimes \widehat{B}_j, \end{aligned}$$

where $\widehat{B}_j$ can be any Hermitian operators acting on the environmental Hilbert space. For the conventional spin-boson model (16), the environmental operators are taken to be $\widehat{B}_\uparrow =-\widehat{B}_\downarrow =\sum _{\vec {k}}\hbar (g_{\vec {k}}\hat{b}_{\vec {k}}^\dagger +g_{\vec {k}}^*\hat{b}_{\vec {k}})$. We slightly generalize the conventional model (16) to a biased one and replace the environmental operators with

23 $$\begin{aligned} \widehat{B}_j=\sum _{\vec {k}}\hbar (g_{j,\vec {k}}\hat{b}_{\vec {k}}^\dagger +g_{j,\vec {k}}^*\hat{b}_{\vec {k}}). \end{aligned}$$

Note that the coupling constants $g_{j,\vec {k}}$’s have *j*-dependence and would vary with *j*.

**Figure 4:**
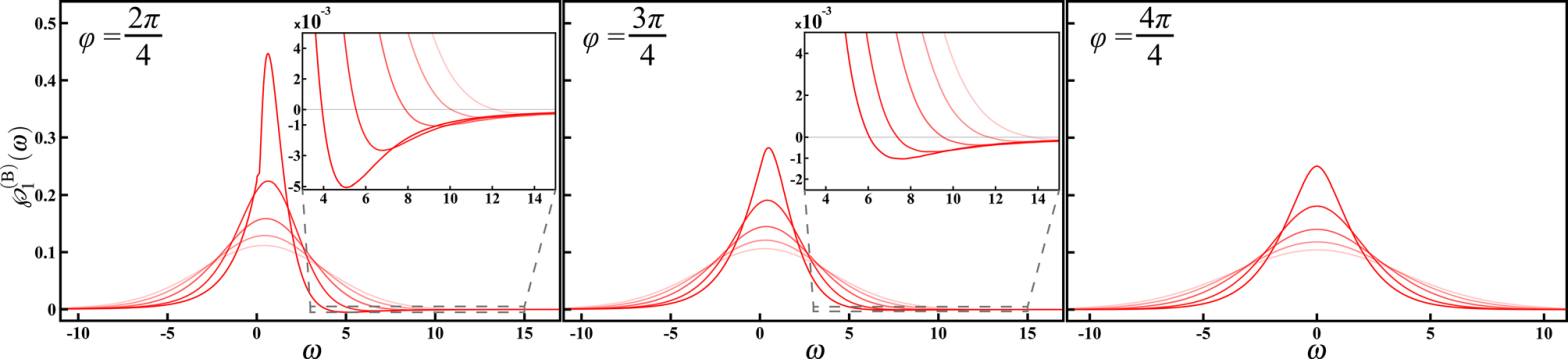
The CHER of the biased spin-boson model. We investigate the nonclassical effects of the relative phase $\varphi$ between the two coupling constants under the balanced condition. In the left ($\varphi =2\pi /4$) and middle ($\varphi =3\pi /4$) panels, we can see shallow, and stretched, negative wings, which are further enlarged in the insets. As *T* increases, the CHER broadens, and the negative wing gradually fades away. As $\varphi$ approaching $\pi$ (from left, middle, to right panels), it recovers the conventional model, and, consequently, the CHER for $\varphi =\pi$ is positive. In these plots, we have assumed $\omega _{\rm {c}}=1$, $k_{\rm {B}}/\hbar =1$, and increasing *T* from 0 to 2 (decreasing opacity).

In the interaction picture (with respect to $\widehat{H}_{\rm S}+\widehat{H}_{\rm E}$), the total system evolves according to $\widehat{U}^{\rm I}(t)=\mathcal {T}\left\{ \exp \left[ (-i/\hbar )\int _0^t\widehat{H}_{\rm I}(\tau )d\tau \right] \right\}$, where $\mathcal {T}$ is the time-ordering operator. To investigate the nonclassical effects caused by the relative phase between the coupling constants, we assume, for simplicity, a balanced condition with finite relative phase, i.e., $g_{\downarrow ,\vec {k}}=g_{\uparrow ,\vec {k}}e^{i\varphi }$. This model can also be solved analytically and the qubit pure dephasing is characterized by the dephasing factor

24 $$\begin{aligned} \phi ^{({\rm B})}(t)=\exp \left[ -i\vartheta ^{({\rm B})} (t)-\Phi ^{({\rm B})}(t)\right] , \end{aligned}$$

where $\vartheta ^{({\rm B})}(t)=2{\rm sign}(t)\sin \varphi \int _0^\infty [\mathcal {J}(\omega )/\omega ^2](1-\cos \omega t)d\omega$ and $\Phi ^{({\rm B})}(t)=2(1-\cos \varphi )\int _0^\infty [\mathcal {J}(\omega )/\omega ^2]\coth (\hbar \omega /2k_{\rm B}T)(1-\cos \omega t)d\omega$. In the expression of $\vartheta ^{({\rm B})}(t)$, we have manually inserted ${\rm sign}(t)$. While this does not affect the pure dephasing dynamics for $t\ge 0$, it ensures that the property $\phi ^{({\rm B})}(-t)=\phi ^{({\rm B})*}(t)$ holds and the CHER $\wp ^{({\rm B})}(\omega )$ is a real function (condition C2). Details can be found in "Methods".

We now revisit the Ohmic spectral density $\mathcal {J}_1(\omega )=\omega \exp (-\omega /\omega _{\rm c})$ at finite temperature *T*, where the dephasing factor is given by

25 $$\begin{aligned} \phi _1^{({\rm B})}(t)=\frac{1}{\left( 1+\omega _{\rm c}^2 t^2\right) ^{(1-\cos \varphi )+i{\rm sign}(t)\sin \varphi }} \prod _{n=1}^\infty \left[ 1+\left( \frac{\omega _{\rm c}k_{\rm B}T}{k_{\rm B}T +n\hbar \omega _{\rm c}}\right) ^2t^2\right] ^{-2(1-\cos \varphi )}. \end{aligned}$$

The numerical results of the corresponding CHER $\wp _1^{({\rm B})}(\omega )=(2\pi )^{-1}\int _{-\infty }^\infty \phi _1^{({\rm B})}(t)\exp (i\omega t)dt$ are shown in Fig. 4 with $\omega _{\rm c}=1$, $k_{\rm B}/\hbar =1$, and an increasing *T* from 0 to 2 (decreasing opacity). In the left ($\varphi =2\pi /4$) and middle ($\varphi =3\pi /4$) panels of Fig. 4, we can see a shallow, and stretched, negative wing for each curve. The insets further zoom in to the negative wings. This is a signature of the nonclassical trait of this biased model, clearly indicating the emergence of nonclassical correlations between the qubit and it environment. As *T* increases from 0 to 2 (decreasing opacity), the negative wing gradually fades away and the CHER becomes broader and lower, in line with the usual intuition that the thermal fluctuations are detrimental to quantum nature. On the other hand, for the case of $\varphi =\pi$, all the formulae reduce to the conventional ones; consequently, the CHER for $\varphi =\pi$ is positive and the right panel of Fig. 4 reproduces the one of Fig. 3.

### Uniqueness of CHER for pure dephasing

Through the above examples, one may be convinced of the uniqueness of the CHER for qubit pure dephasing, as $\wp (\omega )$ is obtained from the Fourier inverse transform of dephasing factor $\phi (t)$ via Eq. (18). Nevertheless, whenever one considers the case of higher dimensional pure dephasing, this becomes problematic as Eq. (18) is never applicable for higher dimensional cases.

In fact, the uniqueness still holds, with respect to diagonal member Hamiltonian operators, even if one considers the case of any dimensional pure dephasing. More precisely, it has been proven^48^ that, given any dimensional pure dephasing, there exists a unique CHER, encapsulated within a HE of diagonal member Hamiltonian operators, satisfying Eq. (12). The spirit of the proof is based on the abelian nature of diagonal member Hamiltonian operators in the HE and provides further insights into the algebraic structure behind the CHER.

The Fourier transform in Eq. (2) integrates over full real numbers, which forms an abelian group with respect to multiplication. However, the unitary group generated by Hermitian operators fails to be abelian. This renders the general solution to Eq. (12) highly nontrivial. Therefore, the closely related problem of random-unitary decomposition can only be tackled numerically ^66^.

To circumvent this issue, we may restrict ourselves to an abelian one. Accordingly, if we consider the group $\mathcal {G}$ in Eq. (12) to be generated by a maximally abelian subalgebra, i.e., the Cartan subalgebra of $\mathfrak {su}(n)$, then several intuitive algebraic properties are inherited from the conventional Fourier transform, including the one-to-one correspondence between $\wp$ and $\mathcal {E}_t$. Consequently, under the framework of Cartan subalgebra, we can explicitly deal with the bijective correspondence between pure dephasing and the CHER with respect to diagonal member Hamiltonian operators. In other words, the uniqueness of CHER for pure dephasing straightforwardly follows, with respect to diagonal member Hamiltonian operators.

### Multiple representations for unital dynamics

Based on the above algebraic insights into the CHER, it is straightforward to deduce the breakdown of the uniqueness of the CHER whenever one goes beyond pure dephasing. To examine this, we explain how to construct multiple $\wp$ satisfying Eq. (12) simultaneously for a given qubit unital dynamics $\mathcal {E}_t$, which may either result theoretically from solving certain master equation or experimentally from process tomography raw data.

Since now we are working with $\mathfrak {u}(2)=\mathfrak {u}(1)\oplus \mathfrak {su}(2)$, the generators are explicitly chosen to be identity $\widehat{I}$ and Pauli matrices ${\hat{\sigma }}_j$. The matrix elements of the dynamical linear map $\mathcal {E}_t^{(\tilde{\sigma })}$ on the left hand side of Eq. (12) are determined according to

26 $$\begin{aligned} \left[ \mathcal {E}_t^{(\tilde{\sigma })}\right] _{j,k}= \frac{1}{2}{\rm Tr}\left[ {\hat{\sigma }}_j\cdot \mathcal {E}_t\{{\hat{\sigma }}_k\}\right] . \end{aligned}$$

On the other hand, according to the commutation relation $[{\hat{\sigma }}_j,{\hat{\sigma }}_k]=\varepsilon _{jkl}i2{\hat{\sigma }}_l$, the Pauli matrices in the adjoint representation are written as

27 $$\begin{aligned} \tilde{\sigma }_x=\left[ \begin{array}{c|ccc} 0 &{} 0 &{} 0 &{} 0 \\ \hline 0 &{} 0 &{} 0 &{} 0 \\ 0 &{} 0 &{} 0 &{} -i2\\ 0 &{} 0 &{} i2&{} 0 \end{array} \right] , \tilde{\sigma }_y=\left[ \begin{array}{c|ccc} 0 &{} 0 &{} 0 &{} 0 \\ \hline 0 &{} 0 &{} 0 &{} i2 \\ 0 &{} 0 &{} 0 &{} 0 \\ 0 &{} -i2&{} 0 &{} 0 \end{array} \right] , \tilde{\sigma }_z=\left[ \begin{array}{c|ccc} 0 &{} 0 &{} 0 &{} 0 \\ \hline 0 &{} 0 &{}-i2&{} 0 \\ 0 &{} i2&{} 0 &{} 0 \\ 0 &{} 0 &{} 0 &{} 0 \end{array} \right] . \end{aligned}$$

Note that $\widetilde{I}=0$ as $[\widehat{I},{\hat{\sigma }}_j]=0$, $\forall ~j$. For any $\widehat{H}_\lambda =(\lambda _0\widehat{I}+\varvec{\lambda }\cdot \hat{\varvec{\sigma }})/2\in \mathfrak {u}(2)$, its adjoint representation reads

28 $$\begin{aligned} \widetilde{H}_\lambda =(\varvec{\lambda }\cdot \tilde{\varvec{\sigma }})/2=\left[ \begin{array}{c|ccc} 0 &{} 0 &{} 0 &{} 0 \\ \hline 0 &{} 0 &{} -i\lambda _z &{} i\lambda _y \\ 0 &{} i\lambda _z &{} 0 &{} -i\lambda _x\\ 0 &{} -i\lambda _y &{} i\lambda _x&{} 0 \end{array} \right] . \end{aligned}$$

Then the unitary operator in the adjoint representation on the right hand side of Eq. (12) can be expressed as

29 $$\begin{aligned} e^{-i(\varvec{\lambda }\cdot \tilde{\varvec{\sigma }}t)/2}=\left[ \begin{array}{c|ccc} 1 &{} 0 &{} 0 &{} 0 \\ \hline 0 &{} &{} &{} \\ 0 &{} &{} \widetilde{\mathcal {U}}_t &{} \\ 0 &{} &{} &{} \\ \end{array}\right] , \end{aligned}$$

where $\widetilde{\mathcal {U}}_t$ is a $3\times 3$ orthogonal matrix. Its elements are given in "Methods" with the change of parameters to spherical coordinate $\lambda _x=\omega \sin \theta \cos \psi$, $\lambda _y=\omega \sin \theta \sin \psi$, and $\lambda _z=\omega \cos \theta$. After determining the matrix elements on both sides, Eq. (12) implies 9 equations governing $\wp (\omega ,\theta ,\psi )$:

30 $$\begin{aligned} \left[ \mathcal {E}_t^{(\tilde{\sigma })}\right] _{j,k}=\int \int _0^\infty \wp (\omega ,\theta ,\psi )\left[ \widetilde{\mathcal {U}}_t\right] _{j,k}\omega ^2 d\omega d\Omega . \end{aligned}$$

As shown in "Methods", each $\left[ \widetilde{\mathcal {U}}_t\right] _{j,k}$ can also be expanded in terms of spherical harmonics ${\rm Y}_{l,m}(\theta ,\psi )$ in real form with $l\le 2$. We can therefore expand $\wp (\omega ,\theta ,\psi )$ as well and divide the expansion into two types according to the index *l*

31 $$\begin{aligned} \wp (\omega ,\theta ,\psi )=\sum _{l\le 2,m}\wp _{l,m}(\omega ){\rm Y}_{l,m}(\theta ,\psi ) +\sum _{l\ge 3,m}\wp _{l,m}(\omega ){\rm Y}_{l,m}(\theta ,\psi ). \end{aligned}$$

There are 9 terms in the first type with $l\le 2$; therefore $\wp _{l,m}(\omega )$ can be solved with Eq. (30). On the other hand, the second type with $l\ge 3$ has no contribution to Eq. (30) due to the orthogonality of ${\rm Y}_{l,m}(\theta ,\psi )$.

These observations also imply that, if two CHERs only differ in the second type with $l\ge 3$, i.e., $\wp _1-\wp _2=\sum _{l\ge 3,m}\Delta \wp _{l,m}(\omega ){\rm Y}_{l,m}(\theta ,\psi )$, then the two CHERs simultaneously satisfy Eq. (30), as well as Eq. (12). Consequently, we can draw the conclusion of the breakdown of the uniqueness of the CHER beyond pure dephasing.

### Experimental proposal

Finally, in order to underpin the practical viability of the CHER theory, here we present a promising experimental proposal based upon free induction decay (FID) measurement of the negatively charged nitrogen-vacancy (NV$^{-}$) center in a diamond^67–69^. The NV$^{-}$ center has an electron spin-1 triplet as its ground states, with a zero-field splitting $D=2.87$ GHz between $m_{\rm S}=0$ and $m_{\rm S}=\pm 1$. The degeneracy between $m_{\rm S}=\pm 1$ can be lifted by applying an external magnetic field, allowing selective microwave (MW) excitation of a single spin transition $|0\rangle \leftrightarrow |1\rangle$ and forming a logical qubit. Due to strong binding between carbon atoms in the diamond lattice, the electron spin coherence time is not limited by the spin-lattice interactions. Therefore, many interesting quantum effects can be observed even at room temperature. Rather, the spin qubit operation is subjected to noise mainly from the nuclear spin bath formed by the $^{13}$C isotope (1.1$\%$ natural abundance), leading to a spin qubit relaxation time $T_1$ in the order of milliseconds^70,71^ and a dephasing time $T_2^*$ of microseconds^72,73^. Several techniques have been developed for prolonging the coherence time, e.g., isotopic purification^74^ and nuclear spin polarization^75,76^.

**Figure 5:**
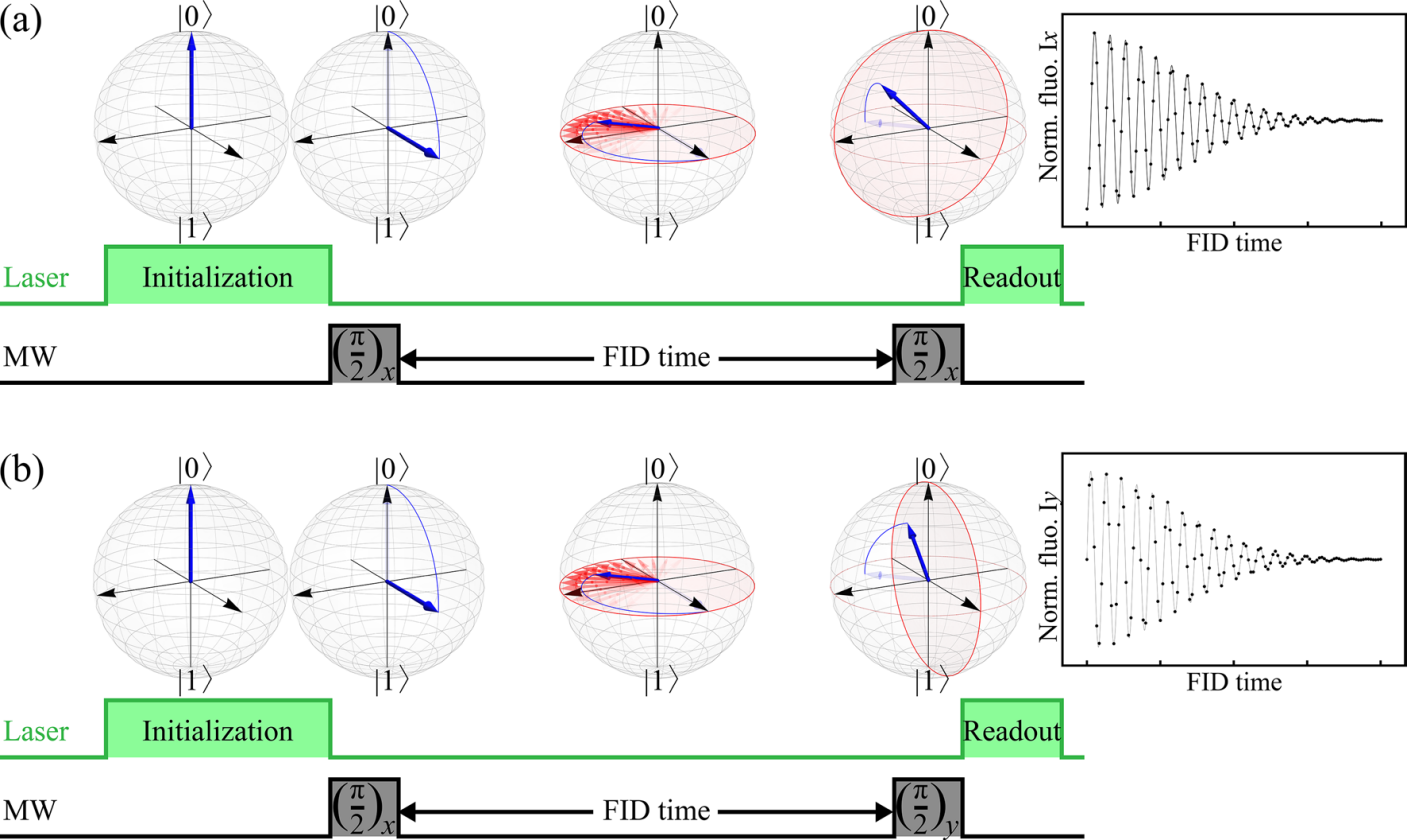
The proposed experimental realization is composed of two FID signals. (**a**) After the electron spin initialization to $|0\rangle$ by a 532-nm green laser, the standard Ramsey pulse sequence consists of two MW $(\pi /2)_x$ pulses separated by an FID time period, during which the spin qubit pure dephasing takes place. The final laser is used to detect the remaining population in the $|0\rangle$ state and the normalized fluorescence ${\rm I}_x(t)$ is recorded. (**b**) To completely reconstruct the phase information, we need to measure the signal along the other orthogonal axis. A variation of the standard Ramsey pulse sequence is adopted by replacing the second MW pulse with $(\pi /2)_y$ and the signal ${\rm I}_y(t)$ is recorded. Then the dephasing factor $\phi (t)$ and the CHER $\wp (\omega )$ can be determined accordingly.

To experimentally reconstruct the dynamical linear map $\mathcal {E}_t^{(\widehat{L})}$ in Eq. (12), one should, in principle, perform the quantum process tomography experiment to gather necessary information on the qubit dynamics. Fortunately, due to the three-order difference between $T_1$ and $T_2^*$, as well as the unique optical properties of the NV$^{-}$ center, the qubit dynamics can be well approximated by pure dephasing. Meanwhile, an appropriate variation of the standard Ramsey pulse sequence shown in Fig. 5 is sufficient for our purpose, circumventing the burden of performing the standard quantum process tomography experiment.

Figure 5a shows the standard Ramsey pulse sequence, beginning with an electron spin initialization to $|0\rangle$ by a 532-nm green laser. A successive MW $(\pi /2)_x$ pulse creates a superposition state and, consequently, turns on the interaction with the nuclear spin bath. The qubit will then undergo an FID process for a period (FID time). The second MW $(\pi /2)_x$ pulse converts the phase information into population. The final 532-nm green laser pumping is used to detect the remaining population in the $|0\rangle$ state and the signal is recorded as the normalized fluorescence ${\rm I}_x(t)$. To completely reconstruct the phase information, an additional measurement is necessary. As shown in Fig. 5b, the second MW pulse is replaced by $(\pi /2)_y$ to extract the phase information along the other orthogonal axis and the signal is recorded as ${\rm I}_y(t)$. The dephasing factor is then determined by the two measured fluorescence signals according to

32 $$\begin{aligned} \phi (t)=-\left[ 2{\rm I}_x(t)-1\right] +i\left[ 2{\rm I}_y(t)-1\right] . \end{aligned}$$

The Fourier transform (18) leads to the resulting $\wp (\omega )$. We stress that this proposed experimental setup is fully compatible with present-day techniques. Finally, it is worth noting that, the entanglement between the electron spin and its $^{13}$C nuclear spin bath established during the FID procedure can be detected with local operations focussing exclusively on the electron spin^65^. This locally detectable entanglement constitutes the origin of nonclassicality in the CHER theory. Our experimental proposal provides further protocol to estimate the presence of the entanglement.

## Discussions and conclusion

In summary, we have elaborated the theoretical foundation for the notion of CHER within a unified framework in detail, as well as its geometrical interpretation in terms of random phase model. Through the Fourier transform on group formalism, it is clearer that CHER plays the role of being the representation of a unital dynamics in the frequency domain. Meanwhile, along with the investigation into the system–environment correlations, we have also discussed its capability of characterizing the nonclassical treats of dephasing dynamics.

Nonclassical distribution over phase space for a state is extensively studied, particularly in the field of quantum optics with the Wigner function^40^ or the Glauber-Sudarshan *P* representation^41,42^. It should be pointed out that similar notion of process nonclassicality has attracted increasing research interests and alternative definitions based on monitoring certain nonclassical traits of the states have been discussed^77–80^ in recent years. Nevertheless, rather than a specified quantum state, we focus on the dynamical processes. More insights can be provided with some further comparative studies.

With several examples, we have also shown generically that the increasing environmental temperature will broaden the CHERs and diminish the nonclassical traits. This is in agreement with the common intuition that the thermal fluctuations are detrimental to the quantum nature. Additionally, we have also demonstrated the transition between Markovianity and non-Markovianity by varying the Ohmicity of the spectral density and the environmental temperature. It is known that non-Markovianity may result from statistical mixture of random unitary^50,66,81,82^ or, more generally, some other dynamical processes^83,84^. Our results suggest that there are discrepancies between the nonclassicality and non-Markovianity, which raise the question of the true quantum non-Markovianity^85^. Notably, as the aforementioned transition phenomenon is even manifest from the shape of CHERs, this may stimulate the development of the techniques of probe for the properties of ambient environments^86–90^.

Furthermore, we have also discussed the uniqueness of CHER for pure dephasing with respect to the Cartan subslgebra of $\mathfrak {su}(n)$. However, when going beyond pure dephasing, the uniqueness breaks down as a result of the analysis on the underlying algebraic structure. To explicitly show the breakdown of uniqueness and explain how to construct multiple CHER beyond pure dephasing, we have considered a general unital dynamics. If two CHERs only differ in the spherical harmonics of an order larger than 3 ($l\ge 3$), the two CHERs represent the same unital dynamics. Interestingly, the question of how to define a principal one among multiple CHERs for unital dynamics can be intriguing. These unresolved points could be seminal and stimulate further studies from many different aspects in the future.

Finally, we have also proposed a promising experimental setup to explain how to realize our CHER theory with measured signals. As the NV$^-$ center possesses several prominent properties and can work under ambient conditions, many techniques have been extensively developed. Therefore, the NV$^-$ center is widely adopted as the testbed of fundamental quantum physics and advanced quantum technologies, e.g., Refs.^91–93^. We propose to utilize this mature platform, along with an appropriate variation of the pulse sequence, and elucidate how to reconstruct the dephasing factor $\phi (t)$ and the resulting CHER $\wp (\omega )$ from the measured fluorescence signals ${\rm I}_x(t)$ and ${\rm I}_y(t)$.

## Methods

### Bochner’s theorem

To prove Proposition 1 in the main text, we need to utilize several mathematical supplements, including the Bochner’s theorem^60^. We first recall the definition of positive definiteness:

#### Definition 2

A function $f:\mathbb {R}\rightarrow \mathbb {C}$ is called to be positive definite if it satisfies

33 $$\begin{aligned} \sum _{j,k}f\left( t_j-t_k\right) z_jz_k^*\ge 0 \end{aligned}$$

for any finite number of pairs $\{(t_j,z_j)|t_j\in \mathbb {R},z_j\in \mathbb {C}\}$.

We stress that the notion of positive definiteness is very different from a positive function since a positive function may not necessarily be positive definite and *vice versa*. Instead, it is equivalent to the positive semidefiniteness of an Hermitian matrix $\left[ f(t_j-t_k)\right] _{j,k\in \mathcal {S}}$ formed by collecting function values $f(t_j-t_k)$ in accordance with certain set of indices $\mathcal {S}$. Particularly, the function *f* in Definition 2 can even be complex. We then find that $\phi (t)$ in Eq. (17) is positive definite^47^, as stated in the following lemma:

#### Lemma 3

*Suppose that the dephasing factor* (17) *defines a CP qubit pure dephasing dynamics. If we further assume that* $\Phi (t)$ *is even and* $\phi (-t)=\phi (t)^*$, *then* $\phi (t)$ *defined on* $\mathbb {R}$ *is positive definite.*

Now we are ready to introduce the Bochner’s theorem. The following expression facilitates our sequential discussions:

#### Theorem 4

(Bochner’s theorem) *A function* $f:\mathbb {R}\rightarrow \mathbb {C}$ *is the Fourier transform of unique positive measure with density function* $\wp$ *if and only if* *f* *is continuous and positive definite.*

Combining above, we can conclude that $\phi (t)$ in Eq. (17) is positive definite and, consequently, is the Fourier transform of certain legitimate probability distribution $\wp (\omega )$, an analog to that in Eq. (2). These results are summarized in Proposition 1 in the main text.

### Vacuum and thermal contributions to the dephasing factor

As discussed in the main text, the qubit pure dephasing reduced from the spin-boson mdoel (16) is characterized by the dephasing factor $\phi (t)=\exp [-i\omega _0t-\Phi (t)]$ with $\Phi (t)=4\int _0^\infty [\mathcal {J}(\omega )/\omega ^2]\coth (\hbar \omega /2k_{\rm B}T)(1-\cos \omega t)d\omega$. It is convenient to split it into the vacuum and thermal parts^1^ as $\Phi (t)=\Phi ^{({\rm vac})}(t)+\Phi ^{({\rm th})}(t)$, where

34 $$\begin{aligned} \Phi ^{({\rm vac})}(t)=4\int _0^\infty \frac{\mathcal {J}(\omega )}{\omega ^2}(1-\cos \omega t)d\omega \end{aligned}$$

and

35 $$\begin{aligned} \Phi ^{({\rm th})}(t)=4\int _0^\infty \frac{\mathcal {J}(\omega )}{\omega ^2}\left[ \coth \left( \frac{\hbar \omega }{2k_{\rm B}T}\right) -1\right] (1-\cos \omega t)d\omega . \end{aligned}$$

It can be seen that $\Phi ^{({\rm vac})}(t)$ is independent of temperature and corresponds to the contribution in the zero-temperature limit. The impact of thermal fluctuations is taken into account by the thermal contribution $\Phi ^{({\rm th})}(t)$.

### Ohmic spectral density

Considering the case of Ohmic spectral density $\mathcal {J}_1(\omega )=\omega \exp (-\omega /\omega _{\rm c})$, the vacuum contribution (34) can be obtained with the help of series expansion as

36 $$\begin{aligned} \Phi _1^{({\rm vac})}(t)= & {} 4\int _0^\infty \frac{1-\cos \omega t}{\omega }e^{-\frac{\omega }{\omega _{\rm c}}}d\omega \nonumber \\= & {} 4\sum _{n=1}^\infty \int _0^\infty \frac{(-1)^{n-1}}{2n!}\omega ^{2n-1}t^{2n}e^{-\frac{\omega }{\omega _{\rm c}}}d\omega \nonumber \\= & {} 4\sum _{n=1}^\infty \frac{(-1)^{n-1}}{2n}\omega _{\rm c}^{2n}t^{2n}=2\ln \left( 1+\omega _{\rm c}^2t^2\right) . \end{aligned}$$

With the help of the expansion $\coth x=1+2\sum _{n=1}^\infty \exp (-2nx)$, the thermal contribution (35) can be easily obtained in a similar manner:

37 $$\begin{aligned} \Phi _1^{({\rm th})}(t)= & {} 4\int _0^\infty \frac{1-\cos \omega t}{\omega }e^{-\frac{\omega }{\omega _{\rm c}}}\left[ \coth \left( \frac{\hbar \omega }{2k_{\rm B}T}\right) -1\right] d\omega =\sum _{n=1}^\infty 4\int _0^\infty \frac{1-\cos \omega t}{\omega }2e^{-\left( \frac{1}{\omega _{\rm c}}+\frac{n\hbar }{k_{\rm B}T}\right) \omega }d\omega \nonumber \\= & {} 4\sum _{n=1}^\infty \ln \left[ 1+\left( \frac{\omega _{\rm c}k_{\rm B}T}{k_{\rm B}T+n\hbar \omega _{\rm c}}\right) ^2t^2\right] . \end{aligned}$$

Then the dephasing factor is given by $\phi _1(t)=\exp [-\Phi _1^{({\rm vac})}(t)-\Phi _1^{({\rm th})}(t)]$. This reproduces Eq. (19) in the main text.

### Family of super-Ohmic spectral densities

In the example of super-Ohmic spectral densities $\mathcal {J}_s(\omega )=\eta \omega ^s\omega ^{1-s}_{\rm c}\exp (-\omega /\omega _{\rm c})$, the vacuum part can be split into three terms:

38 $$\begin{aligned} \Phi _s^{({\rm vac})}(t)= & {} 4\int _0^\infty \eta \frac{\omega ^{s-2}}{\omega ^{s-1}_{\rm c}}(1-\cos \omega t)e^{-\frac{\omega }{\omega _{\rm c}}}d\omega \nonumber \\= & {} 4\eta \int _0^\infty \frac{\omega ^{s-2}}{\omega ^{s-1}_{\rm c}}e^{-\frac{\omega }{\omega _{\rm c}}}d\omega -2\eta \int _0^\infty \frac{\omega ^{s-2}}{\omega ^{s-1}_{\rm c}}e^{-i\omega t} e^{-\frac{\omega }{\omega _{\rm c}}}d\omega -2\eta \int _0^\infty \frac{\omega ^{s-2}}{\omega ^{s-1}_{\rm c}}e^{i\omega t} e^{-\frac{\omega }{\omega _{\rm c}}}d\omega . \end{aligned}$$

Then, along with the definition of gamma function $\Gamma (s+1)=\int _0^\infty x^s \exp (-x)dx$ and usual calculation, one can obtain Eq. (20) in the main text.

In the case of finite temperature *T*, the thermal part is present and written as

39 $$\begin{aligned} \Phi _s^{({\rm th})}(t)=4\int _0^\infty \eta \frac{\omega ^{s-2}}{\omega ^{s-1}_{\rm c}}(1-\cos \omega t)e^{-\frac{\omega }{\omega _{\rm c}}} \left[ \coth \left( \frac{\hbar \omega }{2k_{\rm B}T}\right) -1\right] d\omega . \end{aligned}$$

By using similar approaches, one can obtain

40 $$\begin{aligned} \Phi _s^{({\rm th})}(t)= & {} 4\eta \sum _{n=1}^\infty \int _0^\infty \frac{\omega ^{s-2}}{\omega ^{s-1}_{\rm c}}(2-e^{-i\omega t}-e^{i\omega t}) e^{-\left( \frac{1}{\omega _{\rm c}}+\frac{n\hbar }{k_{\rm B}T}\right) \omega }d\omega \nonumber \\= & {} 4\eta \sum _{n=1}^\infty \Gamma (s-1) \left[ \frac{2}{\left( 1+n\frac{\hbar \omega _{\rm c}}{k_{\rm B}T}\right) ^{s-1}}-\frac{1}{\left( 1+n\frac{\hbar \omega _{\rm c}}{k_{\rm B}T}+i\omega _{\rm c}t\right) ^{s-1}} -\frac{1}{\left( 1+n\frac{\hbar \omega _{\rm c}}{k_{\rm B}T}-i\omega _{\rm c}t\right) ^{s-1}}\right] \nonumber \\= & {} -4\eta \Gamma (s-1)\left[ 2-\frac{\left( 1+i\omega _{\rm c}t\right) ^{s-1}+\left( 1-i\omega _{\rm c}t\right) ^{s-1}}{\left( 1+\omega _{\rm c}^2t^2\right) ^{s-1}}\right] \nonumber \\&+4\eta \Gamma (s-1)\left( \frac{k_{\rm B}T}{\hbar \omega _{\rm c}}\right) ^{s-1}\left[ 2\zeta \left( s-1,\frac{k_{\rm B}T}{\hbar \omega _{\rm c}}\right) -\zeta \left( s-1,\frac{k_{\rm B}T}{\hbar \omega _{\rm c}}(1+i\omega _{\rm c}t)\right) -\zeta \left( s-1,\frac{k_{\rm B}T}{\hbar \omega _{\rm c}}(1-i\omega _{\rm c}t)\right) \right] , \nonumber \\ \end{aligned}$$

where $\zeta (s,q)=\sum _{n=0}^\infty (q+n)^{-s}$ is the Hurwitz zeta function. Then Eq. (21) is given by $\Phi _s(t)=\Phi _s^{({\rm vac})}(t)+\Phi _s^{({\rm th})}(t)$.

### Biased spin-boson model

We proceed to the biased spin-boson model with interaction Hamiltonian given by Eqs. (22) and (23). In the interaction picture with respect to $\widehat{H}_{\rm S}+\widehat{H}_{\rm E}$, it is written as

41 $$\begin{aligned} \widehat{H}_{\rm I}(t)=\sum _{j=\uparrow ,\downarrow }|j\rangle \langle j|\otimes \widehat{B}_j^{{\rm I}}(t), \end{aligned}$$

where

42 $$\begin{aligned} \widehat{B}_j^{{\rm I}}(t)=\sum _{\vec {k}}\hbar (g_{j,\vec {k}}\hat{b}_{\vec {k}}^\dagger e^{i\omega _{\vec {k}} t} +g_{j,\vec {k}}^*\hat{b}_{\vec {k}}e^{-i\omega _{\vec {k}} t}). \end{aligned}$$

Note that the interaction Hamiltonian do not commute to each other at different time:

43 $$\begin{aligned} \left[ \widehat{H}_{\rm I}(\tau ),\widehat{H}_{\rm I}(\tau ^\prime )\right] =\sum _{j=\uparrow ,\downarrow }|j\rangle \langle j|\otimes \sum _{\vec {k}}-i2\hbar ^2|g_{j,\vec {k}}|^2\sin \omega _{\vec {k}}\left( \tau -\tau ^\prime \right) . \end{aligned}$$

Then the time-ordering $\mathcal {T}$ in the unitary evolution operator $\widehat{U}^{\rm I}(t)=\mathcal {T}\left\{ \exp \left[ (-i/\hbar )\int _0^t\widehat{H}_{\rm I}(\tau )d\tau \right] \right\}$ will play a significant role^1^:

44 $$\begin{aligned} \widehat{U}^{\rm I}(t)= & {} \exp \left[ -\frac{1}{2\hbar ^2}\int _0^t\int _0^\tau [\widehat{H}_{\rm I}(\tau ),\widehat{H}_{\rm I}(\tau ^\prime )]d\tau ^\prime d\tau \right] \exp \left[ -\frac{i}{\hbar }\int _0^t\widehat{H}_{\rm I}(\tau )d\tau \right] \nonumber \\= & {} \sum _{j=\uparrow ,\downarrow }|j\rangle \langle j|\otimes \prod _{\vec {k}}\exp \left[ i|g_{j,\vec {k}}|^2\frac{\omega _{\vec {k}}t-\sin \omega _{\vec {k}}t}{\omega _{\vec {k}}^2}\right] \widehat{D}[g_{j,\vec {k}}\alpha _{\vec {k}}(t)], \end{aligned}$$

where $\widehat{D}[g_{j,\vec {k}}\alpha _{\vec {k}}(t)]=\exp [g_{j,\vec {k}}\alpha _{\vec {k}}(t)\hat{b}_{\vec {k}}^\dagger -g_{j,\vec {k}}^*\alpha _{\vec {k}}^*(t)\hat{b}_{\vec {k}}]$ is the displacement operator and $\alpha _{\vec {k}}(t)=(1-e^{i\omega _{\vec {k}}t})/\omega _{\vec {k}}$.

Now we assume that the initial state $\rho _{\rm T}(0)=\rho _{\rm S}(0)\otimes \rho _{\rm E}(0)$ with $\rho _{\rm E}(0)=\exp [-\widehat{H}_{\rm E}/k_{\rm B}T]/Z$ being an equilibrium state at temperature *T*. Along with the two prescriptions $\widehat{D}[\alpha ]\widehat{D}[\beta ]=\exp [(\alpha \beta ^*-\alpha ^*\beta )/2]\widehat{D}[\alpha +\beta ]$ and $\langle \widehat{D}[\alpha ]\rangle =\exp [-\coth (\hbar \omega /2k_{\rm B}T)|\alpha |^2/2]$, the qubit pure dephasing is characterized by the dephasing factor

45 $$\begin{aligned} \phi ^{({\rm B})}(t)= & {} \left\langle \prod _{\vec {k}}\exp \left[ -i|g_{\downarrow ,\vec {k}}|^2\frac{\omega _{\vec {k}}t-\sin \omega _{\vec {k}}t}{\omega _{\vec {k}}^2}\right] \widehat{D}[-g_{\downarrow ,\vec {k}}\alpha _{\vec {k}}(t)] \prod _{\vec {k}}\exp \left[ i|g_{\uparrow ,\vec {k}}|^2\frac{\omega _{\vec {k}}t-\sin \omega _{\vec {k}}t}{\omega _{\vec {k}}^2}\right] \widehat{D}[g_{\uparrow ,\vec {k}}\alpha _{\vec {k}}(t)]\right\rangle \nonumber \\= & {} \left\langle \prod _{\vec {k}}\exp \left[ -i|g_{\downarrow ,\vec {k}}|^2\frac{\omega _{\vec {k}}t-\sin \omega _{\vec {k}}t}{\omega _{\vec {k}}^2}\right] \exp \left[ i|g_{\uparrow ,\vec {k}}|^2\frac{\omega _{\vec {k}}t-\sin \omega _{\vec {k}}t}{\omega _{\vec {k}}^2}\right] \right. \nonumber \\&\times \left. \exp \left[ -(g_{\downarrow ,\vec {k}}g_{\uparrow ,\vec {k}}^*-g_{\downarrow ,\vec {k}}^*g_{\uparrow ,\vec {k}})\frac{1-\cos \omega _{\vec {k}}t}{\omega _{\vec {k}}^2}\right] \widehat{D}[(g_{\uparrow ,\vec {k}}-g_{\downarrow ,\vec {k}})\alpha _{\vec {k}}(t)] \right\rangle . \end{aligned}$$

In the main text, we have assumed the balanced condition with finite relative phase, $g_{\downarrow ,\vec {k}}=g_{\uparrow ,\vec {k}}e^{i\varphi }$. Then the expression of $\phi ^{({\rm B})}(t)$ can be significantly simplified. Along with the definition of spectral density, $\mathcal {J}(\omega )=\sum _{\vec {k}}|g_{\vec {k}}|^2\delta (\omega -\omega _{\vec {k}})$, we arrive the desired result:

46 $$\begin{aligned} \phi ^{({\rm B})}(t)=\exp \left[ -i\vartheta ^{({\rm B})}(t)-\Phi ^{({\rm B})}(t)\right] , \end{aligned}$$

where $$\vartheta ^{({\rm B})}(t)=2{\rm sign}(t)\sin \varphi \int _0^\infty [\mathcal {J}(\omega )/\omega ^2](1-\cos \omega t)d\omega$$ and $$\Phi ^{({\rm B})}(t)=2(1-\cos \varphi )\int _0^\infty [\mathcal {J}(\omega )/\omega ^2]\coth (\hbar \omega /2k_{\rm B}T)(1-\cos \omega t)d\omega$$.

### The unitary operator in the adjoint representation

The adjoint representation of the unitary operator in Eq. (29) is a $4\times 4$ matrix. However, as we consider qubit unital dynamics, it is of block-diagonalized form with a nontrivial $3\times 3$ block $\widetilde{\mathcal {U}}_t$. Note that $\widetilde{\mathcal {U}}_t$ is a orthogonal matrix generated by $\tilde{\sigma }_j$. Below we explicitly show the matrix elements with the change of parameters to spherical coordinate. Furthermore, each of them can be expanded in terms of spherical harmonics ${\rm Y}_{l,m}(\theta ,\psi )$ in real form with $l\le 2$.

47 $$\begin{aligned} \left[ \widetilde{\mathcal {U}}_t\right] _{11}= & {} \sin ^2\theta \cos ^2\psi +\left( \sin ^2\theta \sin ^2\psi +\cos ^2\theta \right) \cos \omega t \nonumber \\= & {} \frac{\sqrt{4\pi }}{3}{\rm Y}_{0,0}-\sqrt{\frac{4\pi }{45}}{\rm Y}_{2,0}+\sqrt{\frac{4\pi }{15}}{\rm Y}_{2,2} +\left( \frac{\sqrt{16\pi }}{3}{\rm Y}_{0,0}+\sqrt{\frac{4\pi }{45}}{\rm Y}_{2,0}-\sqrt{\frac{4\pi }{15}}{\rm Y}_{2,2}\right) \cos \omega t, \end{aligned}$$

48 $$\begin{aligned} \left[ \widetilde{\mathcal {U}}_t\right] _{21}= & {} \sin ^2\theta \sin \psi \cos \psi (1-\cos \omega t)+\cos \theta \sin \omega t \nonumber \\= & {} \sqrt{\frac{4\pi }{15}}{\rm Y}_{2,-2}(1-\cos \omega t)+\sqrt{\frac{4\pi }{3}}{\rm Y}_{1,0}\sin \omega t, \end{aligned}$$

49 $$\begin{aligned} \left[ \widetilde{\mathcal {U}}_t\right] _{31}= & {} \sin \theta \cos \theta \cos \psi (1-\cos \omega t)-\sin \theta \sin \psi \sin \omega t \nonumber \\= & {} \sqrt{\frac{4\pi }{15}}{\rm Y}_{2,1}(1-\cos \omega t)-\sqrt{\frac{4\pi }{3}}{\rm Y}_{1,-1}\sin \omega t, \end{aligned}$$

50 $$\begin{aligned} \left[ \widetilde{\mathcal {U}}_t\right] _{12}= & {} \sin ^2\theta \sin \psi \cos \psi (1-\cos \omega t)-\cos \theta \sin \omega t \nonumber \\= & {} \sqrt{\frac{4\pi }{15}}{\rm Y}_{2,-2}(1-\cos \omega t)-\sqrt{\frac{4\pi }{3}}{\rm Y}_{1,0}\sin \omega t, \end{aligned}$$

51 $$\begin{aligned} \left[ \widetilde{\mathcal {U}}_t\right] _{22}= & {} \sin ^2\theta \sin ^2\psi +\left( \sin ^2\theta \cos ^2\psi +\cos ^2\theta \right) \cos \omega t \nonumber \\= & {} \frac{\sqrt{4\pi }}{3}{\rm Y}_{0,0}-\sqrt{\frac{4\pi }{45}}{\rm Y}_{2,0}-\sqrt{\frac{4\pi }{15}}{\rm Y}_{2,2} +\left( \frac{\sqrt{16\pi }}{3}{\rm Y}_{0,0}+\sqrt{\frac{4\pi }{45}}{\rm Y}_{2,0}+\sqrt{\frac{4\pi }{15}}{\rm Y}_{2,2}\right) \cos \omega t, \end{aligned}$$

52 $$\begin{aligned} \left[ \widetilde{\mathcal {U}}_t\right] _{32}= & {} \sin \theta \cos \theta \sin \psi (1-\cos \omega t)+\sin \theta \cos \psi \sin \omega t \nonumber \\= & {} \sqrt{\frac{4\pi }{15}}{\rm Y}_{2,-1}(1-\cos \omega t)+\sqrt{\frac{4\pi }{3}}{\rm Y}_{1,1}\sin \omega t, \end{aligned}$$

53 $$\begin{aligned} \left[ \widetilde{\mathcal {U}}_t\right] _{13}= & {} \sin \theta \cos \theta \cos \psi (1-\cos \omega t)+\sin \theta \sin \psi \sin \omega t \nonumber \\= & {} \sqrt{\frac{4\pi }{15}}{\rm Y}_{2,1}(1-\cos \omega t)+\sqrt{\frac{4\pi }{3}}{\rm Y}_{1,-1}\sin \omega t, \end{aligned}$$

54 $$\begin{aligned} \left[ \widetilde{\mathcal {U}}_t\right] _{23}= & {} \sin \theta \cos \theta \sin \psi (1-\cos \omega t)-\sin \theta \cos \psi \sin \omega t \nonumber \\= & {} \sqrt{\frac{4\pi }{15}}{\rm Y}_{2,-1}(1-\cos \omega t)-\sqrt{\frac{4\pi }{3}}{\rm Y}_{1,1}\sin \omega t, \end{aligned}$$

and

55 $$\begin{aligned} \left[ \widetilde{\mathcal {U}}_t\right] _{33}= & {} \cos ^2\theta +\sin ^2\theta \cos \omega t \nonumber \\= & {} \frac{\sqrt{4\pi }}{3}{\rm Y}_{0,0}+\sqrt{\frac{16\pi }{45}}{\rm Y}_{2,0} +\left( \frac{\sqrt{16\pi }}{3}{\rm Y}_{0,0}-\sqrt{\frac{16\pi }{45}}{\rm Y}c_{2,0}\right) \cos \omega t. \end{aligned}$$
