# Publisher Correction: Canonical Hamiltonian ensemble representation of dephasing dynamics and the impact of thermal fluctuations on quantum-to-classical transition

**DOI:** 10.1038/s41598-021-95950-3

**Published:** 2021-09-07

**Authors:** Hong‑Bin Chen, Yueh‑Nan Chen

**Affiliations:** 1 Department of Engineering Science, National Cheng Kung University, Tainan, 70101 Taiwan; 2 Center for Quantum Frontiers of Research and Technology, NCKU, Tainan, 70101 Taiwan; 3 Department of Physics, National Cheng Kung University, Tainan, 70101 Taiwan

Correction to: *Scientific Reports* 10.1038/s41598-021-89400-3, published online 11 May 2021

In the original version of this Article, Yueh-Nan Chen was omitted as a corresponding author. Correspondence and request for materials should be addressed to Hong-Bin Chen and Yueh-Nan Chen. This error has now been corrected in the HTML and PDF versions of the Article.

